# Scientific Education

**Published:** 1902-09-20

**Authors:** 


### The Hospital

A Journal of

The Medical Sciences and Hospital Administration.

Vol. XXXII.—No. 834. Edited by Sir Henry Burdett, K.C.B., and Solomon C. Smith, M.D., M.R.C.P. [September 20, 1902.

### SCIENTIFIC EDUCATION.

The eloquent address in which Professor Dewar has recently called the attention of the British Association for the Advancement of Science, and through that medium the attention of the public, to the present condition of scientific education in England, and to the deplorable consequences which that condition has brought about, is one which should be learnt by heart by every schoolmaster and by every parent in the kingdom. It is not so much that opportunities for scientific training are wanting, as that they are neither fully used nor adequately valued; and the result is, in the Professor's words, that this country "does not possess the diffused education without which the ideas of men of genius cannot fructify beyond the limited scope of an individual." It follows that when a man of genius does arise amongst us, the value of his work, and its possible applications, either in pure science or in the arts, are more readily perceived in foreign countries than among ourselves, and that foreign countries possess a larger proportion of men who, if not capable of originating them, are at least fully capable of turning them to account. The Professor in his discourse referred especially to Germany, and illustrated his remarks chiefly by the history of the aniline-dye industries; but we must recognise that the same superiority exists in France, probably also in Russia, and certainly in Japan, whose *savants*, according to Professor Howes, in his address on Zoology, have not only filled gaps in our growing knowledge, but also, with an acumen deserving the highest praise, have put us right in first principles. It is, in truth, time for us to perceive that the contests to be waged between nations in the future, whether those of the tented field or of the factory and the mart, are likely to be determined by conditions which have never before exerted any potent influence upon events, and that it is necessary, as much for those who desire to retain a foremost place as for those who have only to retain one, that they should learn betimes to adapt themselves to the new circumstances of the coming time, and to grow with them as they are by degrees perfected and developed. The late Sir Andrew Clark was wont to define old age as the state in which the individual had ceased to adjust himself to his environment, and the definition is as true of nations as of individuals. Regarded in this sense, we must no longer regard "old" as a title to be cherished, or at least must show that "old" England is the home of young Englishmen.

There is, perhaps, no direction at present in which it behoves us more carefully to consider our ways than in all that relates to medical education. Ever since the time of Lord Beaconsfield politicians have been accustomed to admit, as a matter of after-dinner speaking, the importance of preserving a high standard of national health; but, prior to Mr. Chamberlain's active support of the labours of the London and the Liverpool Schools of Tropical Medicine, few public men have thought it worth their while to exert themselves, in any effective manner, for the attainment of the end supposed to be desired. Mr. Long has done more to promote the health of dogs than all his colleagues together have done for the members of the human species. And we cannot but think that a great deal of the national inactivity, in this regard, is due to methods of medical education which have left the profession, in many important respects, out of touch with the public to whom they minister, so that the latter are, for the most part, profoundly unconscious of the increasingly scientific aspect of the art of healing, and of the degree in which its proceedings are now the outcome of ascertained principles, rather than of merely empirical conclusions. The more completely this change is effected, and the more completely it is made to write itself upon practice, the more will the physician, as such, be regarded as entitled to that public confidence which is now, to a great extent, withheld from him as a physician, however completely it may be accorded to him as one whose personal merits have had time and opportunity to make themselves known, and who is trusted for himself. If the average doctor were better educated he would probably be in a far better position to speak *ex cathedrâ* by virtue of his calling. It is perhaps, not rash to assume that the art of surgery is not likely to undergo any remarkable new developments during the immediate future, and that the ball now lies at the feet of medicine. The immediate future of medicine, it may be predicted with some confidence, rests on the development of organic chemistry. It would be a matter of some interest to ascertain, among the gentlemen admitted this year to be licentiates of the several colleges of physicians, what proportion are acquainted with even the rudiments of modern organic chemistry, or would be capable of giving an intelligible or intelligent account of its present state and recent progress. All who cannot do so will have to be guided in practice by the knowledge of other people, whose errors, if any, they will be unable to correct by any knowledge of their own.

